# Multi-Objective Optimization of Nitrogen Management for Oat Production in the Agro-Pastoral Ecotone of China Using NSGA-II to Balance Yield, Quality, Nitrogen Use Efficiency and Environmental Sustainability

**DOI:** 10.3390/plants15203127

**Published:** 2026-10-13

**Authors:** Ying Wang, Xue Han, Yanli Zhang, Xiaorong Wu, Lijun Li

**Affiliations:** 1 College of Agronomy, Inner Mongolia Agricultural University, Hohhot 010019, China; nxywy123@imau.edu.cn; 2 Baotou Institute of Agriculture and Animal Husbandry Science and Technology, Baotou 014000, China; 3 Inner Mongolia Academy of Agricultural & Animal Husbandry Sciences, Hohhot 010031, China

**Keywords:** nitrogen management, multi-objective optimization, oat yield, nitrogen use efficiency

## Abstract

Oat is a staple cereal in semi-arid regions and requires eco-friendly nitrogen management to stabilize yield and mitigate environmental risks. A two-year field experiment was conducted to investigate the impacts of nitrogen rate and topdressing timing on oat grain yield, β-glucan yield, economic return, nitrogen agronomic efficiency, and soil mineral nitrogen residue. Jointing stage topdressing at 105–140 kg·ha^−1^ markedly raised grain yield and β-glucan yield. Physiological mechanism analysis revealed that appropriate nitrogen topdressing at the jointing stage effectively maintained a high chlorophyll content and nitrogen balance index by significantly improving the activities of nitrate reductase and glutamine synthetase during the grain-filling stage, thereby promoting the accumulation of dry matter and nitrogen. With the increase in nitrogen application rate and the delay of topdressing time, the soil mineral nitrogen accumulation increased significantly. Furthermore, a multi-objective optimization framework integrating regression models with the NSGA-II algorithm identified the optimal strategy as 140.68 kg·ha^−1^ with topdressing 32 days after emergence. This study constructed a multi-objective optimization system for oat nitrogen application suitable for the agro-pastoral ecotone of northern China, which can provide a theoretical basis and practical methods for the synergistic achievement of high-yield, high-quality oat production and ecological conservation.

## 1. Introduction

The agro-pastoral ecotone in northern China is a semi-arid region, serving as a critical buffer area for agricultural production [1]. However, water scarcity, low soil fertility, and high ecological fragility severely constrain sustainable agricultural development in this region, and insufficient soil nitrogen supply has become a major limiting factor [2]. In local rainfed farmland, farmers tend to overapply nitrogen fertilizer, with the majority of nitrogen applied in one basal dose prior to sowing [3,4,5]. Although such practices can sustain acceptable grain yields, they usually lead to low nitrogen use efficiency and aggravate environmental risks via ammonia volatilization, nitrate leaching, and surface runoff [6]. Therefore, developing nitrogen management strategies to balance productivity, nutrient use efficiency, and environmental sustainability has become a pressing research priority.

China is the largest oat producer in Asia, with approximately 0.7 million ha under cultivation [7]. In addition to its agronomic value, oat is nutritionally unique for its high β-glucan content, a functional dietary fiber that modulates immunity, lowers blood cholesterol, and improves cardiovascular health [8,9]. β-glucan accumulation is highly sensitive to soil nitrogen availability, and higher nitrogen supply promotes nitrogen uptake and assimilation in oats, thereby increasing the β-glucan content and yield of grains [10]. However, excessive nitrogen application can lead to greater soil residual nitrogen accumulation and increased risks of nitrogen loss, reducing nitrogen use efficiency and posing potential environmental threats [6]. Therefore, while improving β-glucan accumulation, nitrogen management should be optimized to achieve a balance between grain quality improvement and environmental sustainability. While recent studies have advanced understanding of oat yield and agronomic traits, research on how nitrogen management regulates β-glucan yield and its trade-offs with grain yield, profitability, and environmental outcomes remains limited.

For oats, previous studies have reported a nitrogen response range of 30–150 kg·ha^−1^ [10,11]. Under favorable soil and water conditions, however, the optimal nitrogen rate can reach 375 kg·ha^−1^ [12]. These findings indicate that the nitrogen response of oats varies substantially across regions and environmental conditions. Besides application rate, fertilization timing is equally important. For wheat, delayed nitrogen topdressing effectively raises yield while reducing losses [13]. For oats, topdressing delayed beyond 39 days after seedling emergence rarely improves grain yield [14]. In rainfed oat fields of northern China, nitrogen is predominantly applied in a single basal application at sowing. Such fertilization practices face constraints in coordinating *N* rates and timings and balancing crop yield, economic return and environmental performance. This highlights an urgent need for optimal nitrogen management strategies that integrate both rate and timing to unravel their interactive effects and to achieve coordinated improvements in yield, quality, economic returns, and ecological sustainability.

Traditional nitrogen fertilizer management strategies are typically oriented toward a single objective such as yield maximization, overlooking the trade-offs among multiple key factors. Genetic algorithms have been applied in the field of crop management owing to their strong global search capability and adaptability [15]. Among them, the Nondominated Sorting Genetic Algorithm II (NSGA-II), first formally proposed by Deb et al. [16], is a well-validated elitist multi-objective evolutionary algorithm. Compared with earlier-generation algorithms, NSGA-II achieves faster convergence speed, superior solution diversity, more robust approximation of the true Pareto front, and lower computational complexity [17]. In agricultural research, NSGA-II has become a critical tool for cropping system optimization, nutrient management, and agroecosystem sustainability assessment. It has been adopted for multi-objective decision-making across various production scenarios: for example, to reconcile corn yield with greenhouse gas emissions under brackish water irrigation [18] and to balance wheat yield against environmental impacts in coupled water–nitrogen management in semi-arid regions [19]. Relevant applied studies have consistently verified the good stability and applicability of the algorithm. In this study, we propose a multi-objective optimization framework for nitrogen management that integrates regression models fitted from field experimental data as the predictive basis with NSGA-II for global optimization, based on the core assumptions of continuous objective functions and sufficient algorithm convergence.

We hypothesize that coupling multiple regression models with the NSGA-II algorithm can be used to identify nitrogen management schemes that simultaneously optimize yield, nutritional quality, and nitrogen use efficiency, while reconciling ecological and economic targets. The objectives of this study are: (1) to explore the synergistic and interactive effects of nitrogen application rate and timing on oat photosynthetic capacity, nitrogen use efficiency, grain yield, nutritional quality, and residual soil nitrogen; (2) to clarify the trade-offs and the underlying mechanisms between oat productivity and environmental sustainability under nitrogen management; and (3) to identify the optimal nitrogen fertilization strategy that simultaneously balances high yield, superior grain quality, high nitrogen use efficiency and low environmental risk via the NSGA-II multi-objective optimization algorithm.

## 2. Results

### 2.1. Grain Yield, β-Glucan Yield and Nitrogen Agronomic Efficiency

The two-year experimental results showed that both nitrogen topdressing timing and nitrogen rate exerted highly significant effects (*p* < 0.01) on oat grain yield, β-glucan yield, and nitrogen agronomic efficiency (NAE) (Table 1). The interaction between nitrogen topdressing timing and nitrogen rate (*N* timing × *N* rate) had a highly significant effect on grain yield in both years (*p* < 0.01). For β-glucan yield, the interaction was highly significant in 2022 (*p* < 0.01). For NAE, the interaction was significant in 2022 (*p* < 0.05). Under the T1 topdressing timing, β-glucan yield increased steadily with increasing nitrogen rate. Compared with N1, the N2–N5 treatments increased β-glucan yield by 9.92–26.50%. Under the T2 and T3 timings, grain yield and β-glucan yield increased initially and then declined with higher nitrogen rates. Compared with N1, the N2, N3, N4, and N5 treatments increased grain yield by an average of 7.74%, 14.95%, 17.35%, and 14.47%, respectively, and β-glucan yield by 10.51%, 18.45%, 21.16%, and 17.30%. Across nitrogen topdressing timings, both grain yield and β-glucan yield increased from T1 to T2, and declined slightly at T3. Among all treatment combinations, the highest grain yield and β-glucan yield were obtained under T2N4, which was not significantly different from those under T2N3 in both years and T2N5 in 2022. For NAE, a consistent decline was observed with increasing nitrogen rate in both years. Compared with N1, the N2–N5 treatments reduced NAE by an average of 15.07–68.99%. NAE showed a trend of initially increasing and then decreasing under different nitrogen topdressing timings. Among all treatment combinations, oat NAE was highest under T2N1 in both years and was significantly higher than other treatments in 2022. The lowest NAE was observed under T3N5 treatments.

### 2.2. Soil Mineral Nitrogen Accumulation Amount

**Figure 1:**
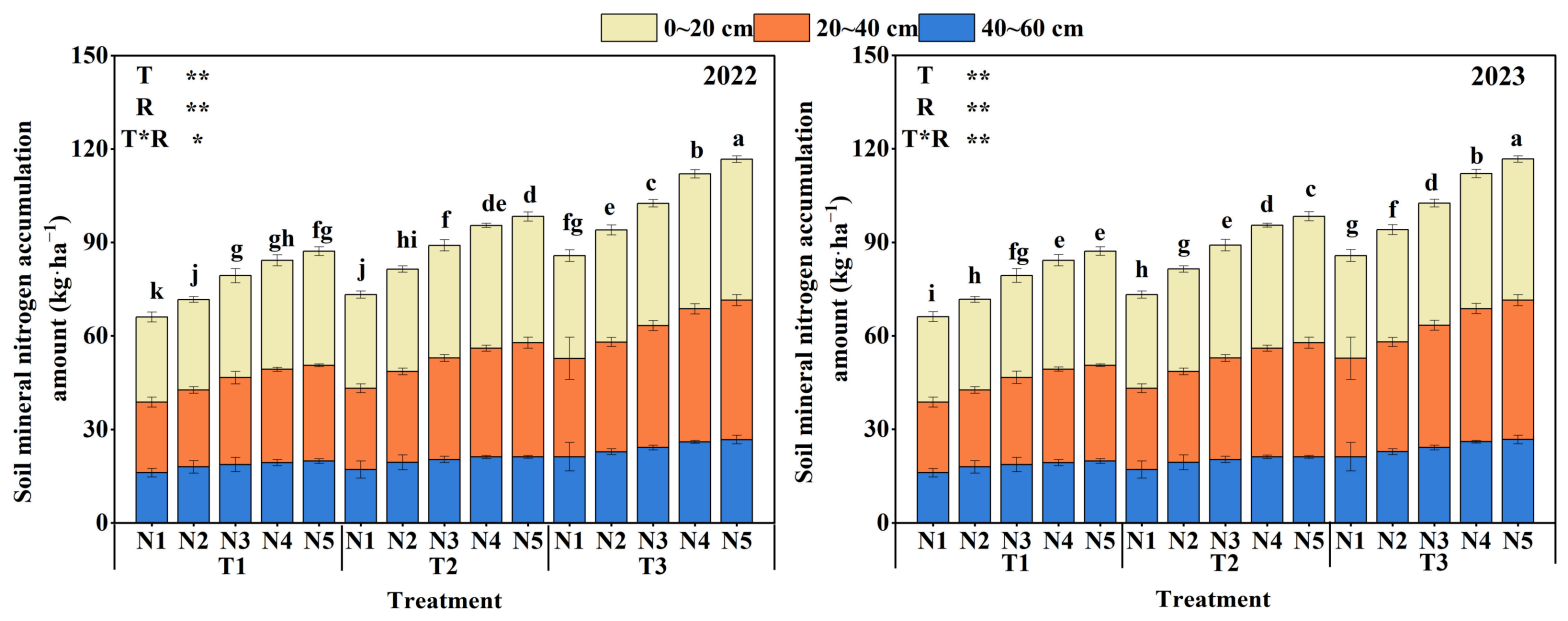
Soil mineral nitrogen accumulation in the 60 cm soil layer under different *N* timing and *N* rate treatments in 2022 and 2023. Different lowercase letters indicate significant differences at *p* < 0.05. * Significant at *p* < 0.05; ** significant at *p* < 0.01. T1, T2, and T3 represent three different topdressing timing treatments, respectively; N1–N5 represent five different nitrogen application rate treatments, respectively.

Both nitrogen topdressing timing and rate had highly significant effects on Soil mineral nitrogen accumulation (SNA) (*p* < 0.01; Figure 1). Their interaction had a highly significant effect in 2023 (*p* < 0.01). With increasing nitrogen rate, SNA in the 0–60 cm soil profile increased progressively; compared with N1, soil mineral nitrogen storage under the N2, N3, N4, and N5 treatments increased by an average of 8.84%, 21.54%, 31.18%, and 36.89%, respectively, across the two years, with differences among rates being statistically significant. Delaying nitrogen topdressing increased soil mineral nitrogen accumulation. Compared with T1, soil mineral nitrogen accumulation under T2 and T3 increased by an average of 10.94% and 25.04%, respectively, across the two years. Overall, the effects of nitrogen topdressing timing and rate were more pronounced in the 0–40 cm soil layer. Among all treatment combinations, soil mineral nitrogen accumulation under T3N5 was significantly higher than that under all other treatments.

### 2.3. Economic Return

Increasing nitrogen application rate significantly raised the total input costs. Compared with N1, the total input of N2–N5 increased by 4.62–18.46%, respectively (Table 2). Results across both years showed that *N* timing and *N* rate had highly significant effects on total revenue and economic return (*p* < 0.01), and their interaction (*N* timing × *N* rate) also exerted significant effects on these two indices (*p* < 0.05). Under the *N* timing, total revenue and economic return increased initially and then declined. Specifically, economic return under T2, N2, N3, N4, and N5 increased by 8.67%, 15.69%, 18.24%, and 13.14% compared with N1. Overall, with delayed nitrogen timing, total revenue and economic return first increased and then decreased. Among all treatment combinations, total revenue and economic return were the highest under T2N4, which were significantly higher than those in other treatments except T2N3 and T2N5. The lowest values were observed under T1N1.

### 2.4. DM Accumulation, N Accumulation, SPAD, NBI, NR Activity and GS Activity

**Figure 2:**
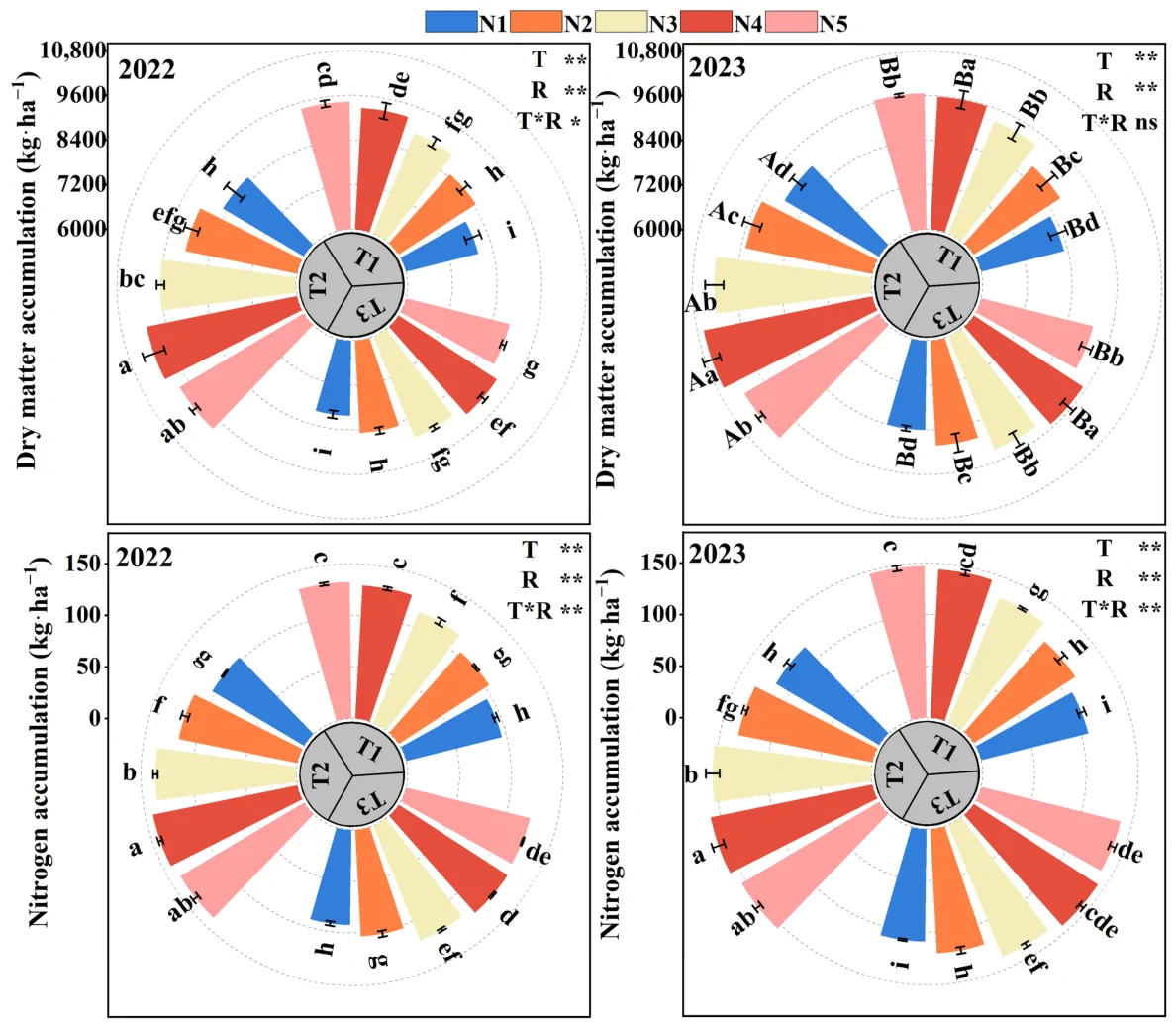
Effects of different *N* timing and *N* rate treatments on dry matter and nitrogen accumulation during 2022–2023. Different lowercase letters indicate significant differences among the 15 treatment combinations at *p* < 0.05 when the *N* timing × *N* rate interaction is significant. When the interaction is not significant, different uppercase letters indicate significant differences for the main effect of *N* timing, and different lowercase letters indicate significant differences for the main effect of *N* rate at *p* < 0.05. * Significant at *p* < 0.05; ** Significant at *p* < 0.01; ns, not significant at *p* > 0.05. T1, T2, and T3 represent three different topdressing timing treatments, respectively; N1–N5 represent five different nitrogen application rate treatments, respectively.

Over the two experimental years, nitrogen topdressing timing and nitrogen rate exerted highly significant effects (*p* < 0.01) on oat dry matter accumulation and nitrogen accumulation (Figure 2). The interaction between nitrogen topdressing timing and nitrogen rate had a significant effect (*p* < 0.05) on DM accumulation in 2022. In contrast, the interaction showed highly significant effects on N accumulation in both years. Under the T1 nitrogen topdressing timing, dry matter and N accumulation increased steadily with increasing nitrogen rates. On average across two years, compared with N1, DM accumulation increased by 5.93%, 11.67%, 15.72%, and 17.23% under N2, N3, N4, and N5, respectively, while N accumulation increased by 9.62%, 22.16%, 35.25%, and 38.38%. Under the T2 and T3 timings, both traits exhibited a quadratic response, increasing up to N4 and declining thereafter. Relative to N1, DM accumulation increased by 5.75–16.16% and N accumulation by 12.53–35.63% under N2N4, but the increments declined at N5. With the delay in nitrogen topdressing timing, both dry matter accumulation and nitrogen accumulation in oat initially increased and then decreased, reaching the maximum under T2 in both years, which were significantly higher than those under T1. Among all treatment combinations, dry matter accumulation and nitrogen accumulation were significantly higher under T2N4 than under all other treatments, except T2N5. In contrast, both parameters were significantly lower under T1N1 than under all other treatments, except T3N1.

**Figure 3:**
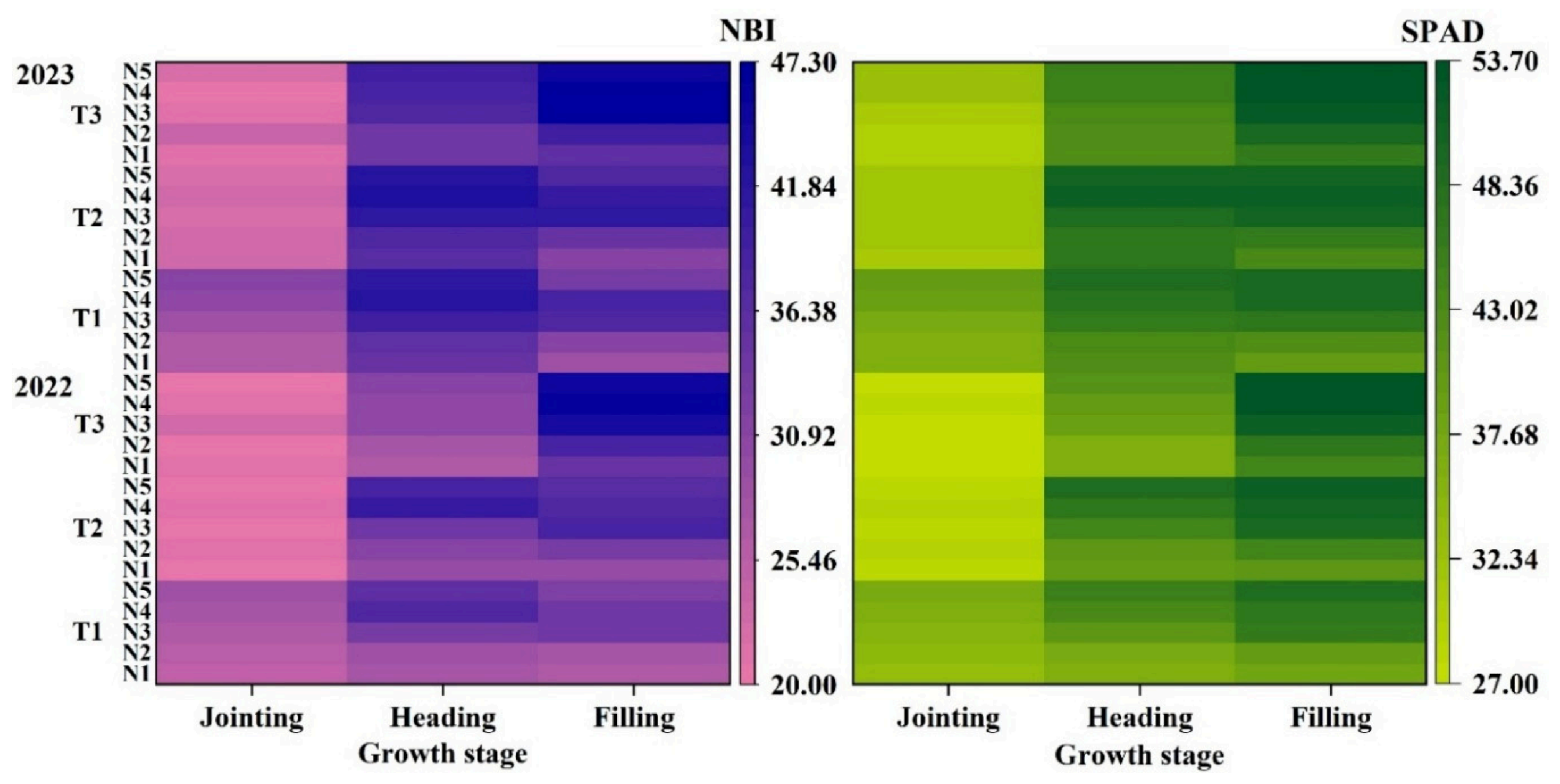
NBI and SPAD values in flag leaves of oat under different treatments during 2022–2023. T1, T2, and T3 represent three different topdressing timing treatments, respectively; N1–N5 represent five different nitrogen application rate treatments, respectively. NBI and SPAD refer to nitrogen balance index and chlorophyll content, respectively.

*N* timing exerted highly significant effects on oat flag leaf SPAD and NBI across all growth stages *(p* < 0.01; Table S1), while *N* rate significantly influenced both indices at heading and filling stages (*p* < 0.01). The *N* timing × *N* rate interaction showed highly significant effects on NBI at the jointing stage in both years (*p* < 0.01), and significantly regulated SPAD and NBI at several other stages. At jointing, delayed N topdressing markedly reduced SPAD and NBI relative to T1 (Figure 3): values decreased by 14.93–23.91% and 19.93–22.58% under T2, and 12.38–28.32% and 17.75–26.80% under T3, respectively. At heading, T2 significantly increased SPAD and NBI by 8.82–10.50% and 4.58–9.59% compared with T1, whereas T3 decreased both indices by 1.16–7.79% and 2.73–20.12%. At filling, SPAD and NBI were significantly higher under T2 than T1 and T3. Delayed N topdressing eliminated significant differences in SPAD and NBI among *N* rates under T2 and T3 at jointing; at other stages, both indices peaked at moderate *N* rates, showing an initial increase followed by a decline with rising N supply. Compared with N1, filling-stage NBI increased by 5.97–11.42%, 25.78–30.79%, 28.87–34.99% and 15.17–30.23% under N2–N5, respectively. Across all treatments, T1N5 yielded the highest SPAD and NBI at jointing, while T2N4 or T2N5 recorded the maximum values at heading and filling stages.

**Figure 4:**
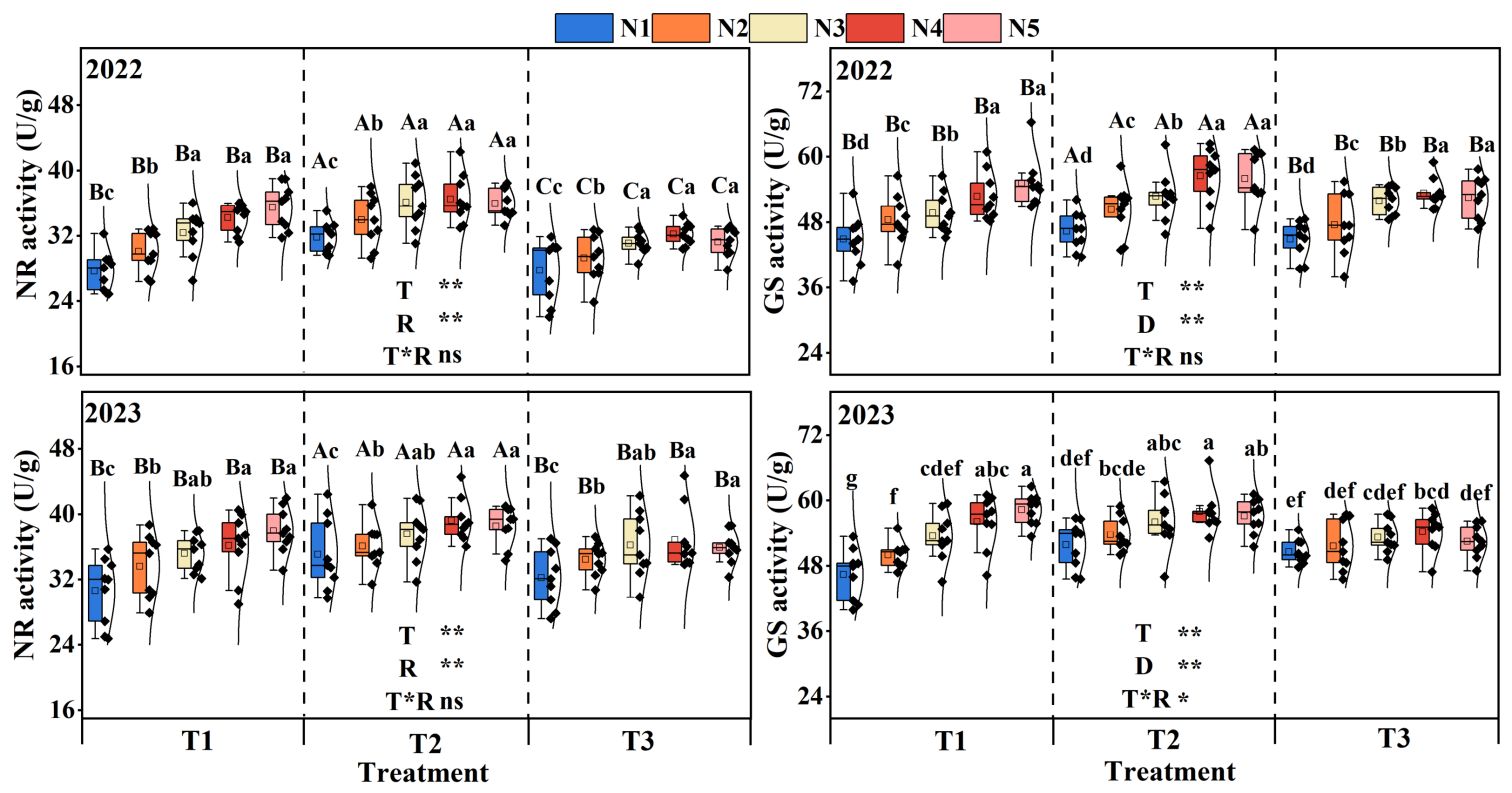
Nitrate reductase (NR) and glutamine synthetase (GS) activities in flag leaves of oat under different treatments during 2022–2023. Different lowercase letters indicate significant differences among the 15 treatment combinations at *p* < 0.05 when the *N* timing × *N* rate interaction is significant. When the interaction is not significant, different uppercase letters indicate significant differences for the main effect of *N* timing, and different lowercase letters indicate significant differences for the main effect of *N* rate at *p* < 0.05. * Significant at *p* < 0.05; ** Significant at *p* < 0.01; ns, not significant at *p* > 0.05. T1, T2, and T3 represent three different topdressing timing treatments, respectively; N1–N5 represent five different nitrogen application rate treatments, respectively.

Across both years, *N* timing and *N* rate exerted highly significant effects on nitrate reductase (NR) and glutamine synthetase (GS) activities (*p* < 0.01), and a significant *N* timing × *N* rate interaction was detected for GS activity in 2023 (*p* < 0.05) (Figure 4). Among N topdressing stages, NR and GS activities were highest under T2, with significant increases of 1.23–13.04% and 1.38–10.42% relative to T1, and 3.36–13.94% and 1.59–8.13% relative to T3, respectively. For *N* rate gradients, both enzyme activities rose continuously with increasing *N* rate under T1, but showed a quadratic increase-then-decrease trend under T2 and T3, peaking at N4. Enzyme activities did not differ significantly between N4 and N5, but were markedly higher than those under N1, N2 and N3. Among all treatment combinations, T2N4 resulted in the highest NR and GS activities; notably, 2023 GS activity under this treatment was only lower than T1N5, T2N3 and T2N5, and significantly higher than all other treatments.

### 2.5. Correlation Analysis of Indicators

**Figure 5:**
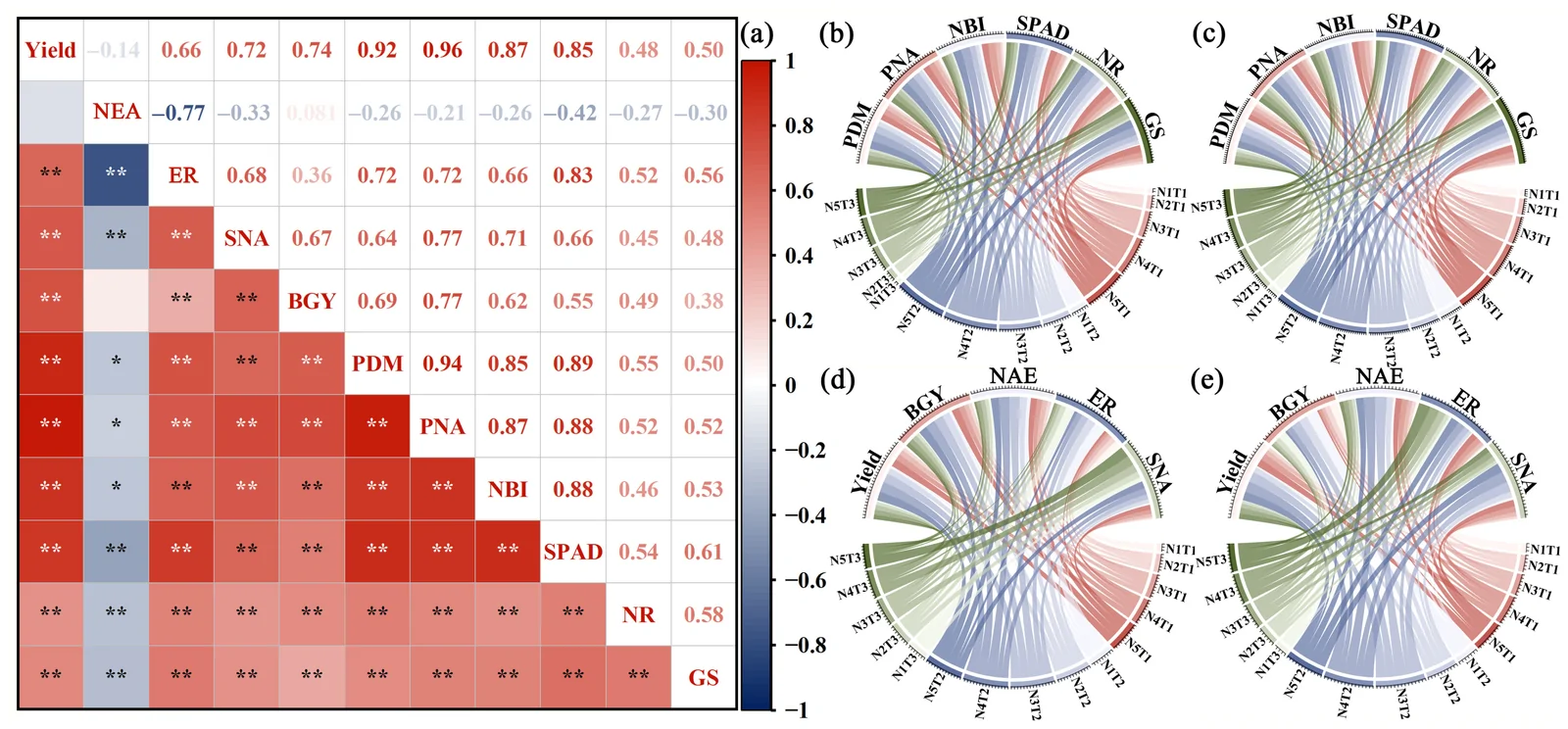
Heatmap of correlations between indicators (**a**). Red represents positive correlation, while blue represents negative correlation. Chord diagrams for each treatment’s physiological indices in 2022 (**b**) and 2023 (**c**) and multi-objective optimization indices in 2022 (**d**) and 2023 (**e**). Note: *, *p* < 0.05; **, *p* < 0.01. Yield: grain yield, NAE: nitrogen agronomic efficiency, ER: economic return, SNA: soil mineral nitrogen accumulation, BGY: β-glucan yield, PDM: plant dry matter accumulation, PNA: plant nitrogen accumulation, NBI: nitrogen balance index, SPAD: chlorophyll content, NR: nitrate reductase activity, GS: glutamine synthetase activity.

**Figure 6:**
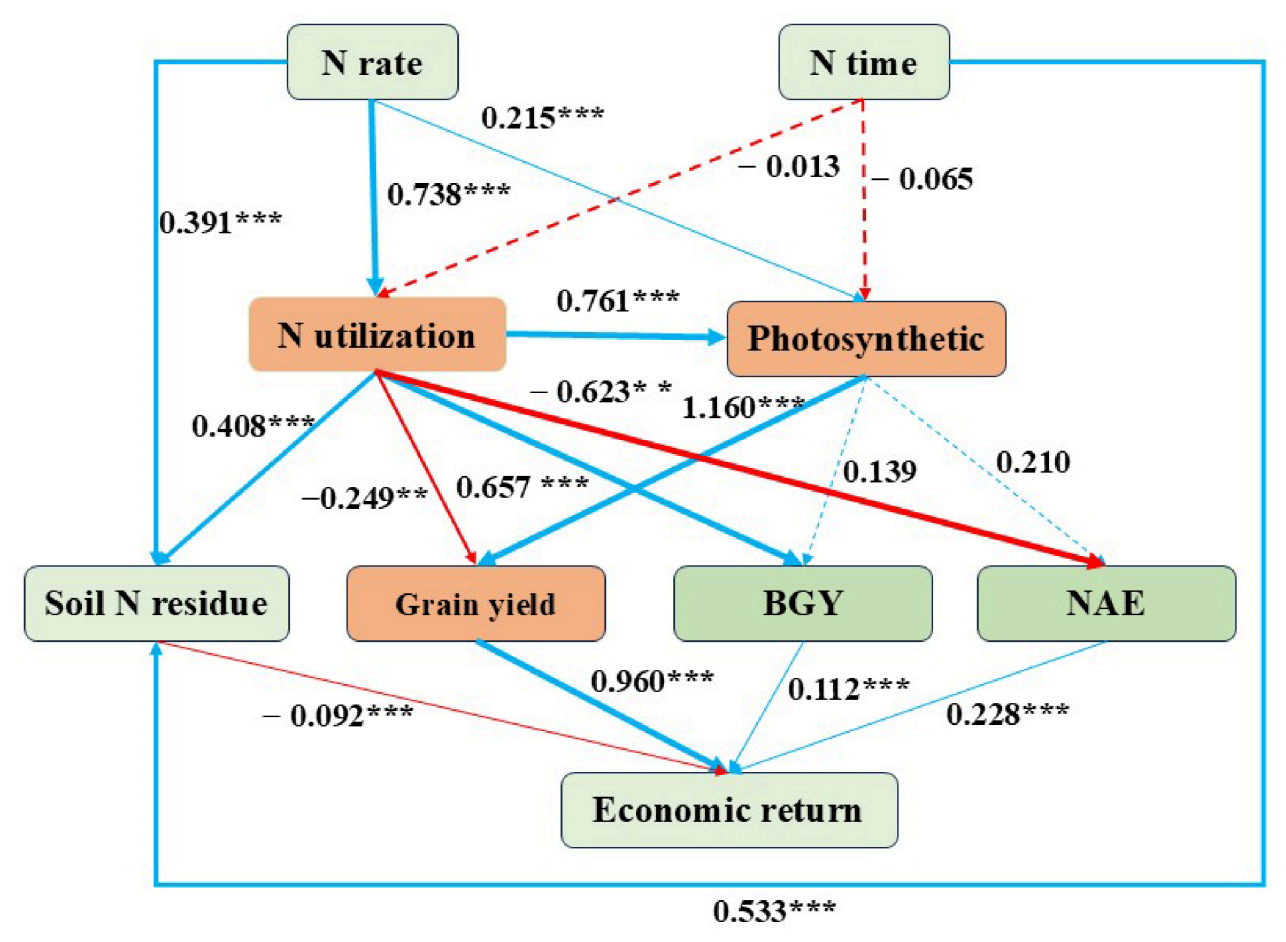
Directed graph demonstrating the partial least squares path model (PLS-PM) for nitrogen rate and topdressing timing effects on N utilization (nitrate reductase activity, glutamine synthetase activity, nitrogen balance index, plant nitrogen accumulation), photosynthetic (plant dry matter accumulation, chlorophyll content), soil N residue, grain yield, BGY (β-glucan yield), NAE (nitrogen agronomic efficiency) and economic return. Each box represents an observed (measured) or latent variable (constructs). Solid lines indicate positive effects, while dashed lines indicate nonsignificant effects (*p* > 0.05), and arrow width is proportional to effect sizes. Asterisks indicate influence levels: ** *p* < 0.01, *** *p* < 0.001. The model was assessed using the Goodness-of-Fit (GoF) statistic, with a GoF value of 0.759.

Correlation analysis revealed that oat physiological traits were highly positively correlated with grain yield, economic return, β-glucan yield, and soil nitrogen residue (*p* < 0.01), indicating their critical roles in yield and quality formation (Figure 5a). In contrast, agronomic nitrogen use efficiency showed significant or highly significant negative correlations with yield and related indicators, suggesting a trade-off between efficiency and output. Chord diagram analysis further showed that the T2N4 treatment exhibited the strongest associations with oat physiological traits and ranked highest in the comprehensive score (Figure 5b–e). PLS-PM analysis demonstrated that nitrogen rate significantly enhanced nitrogen utilization capacity (*p* < 0.001), whereas nitrogen topdressing timing had no significant effect (Figure 6). Nitrogen utilization strongly promoted photosynthetic capacity, which in turn exerted a strong positive effect on yield (*p* < 0.001). However, nitrogen utilization had a negative direct effect on yield, again highlighting the trade-off between efficiency and productivity. Both nitrogen rate and nitrogen topdressing timing, as well as nitrogen utilization, significantly increased soil nitrogen residue (*p* < 0.001), suggesting that excessive nitrogen input elevates the risk of nitrogen surplus. Economic returns were primarily driven by yield and β-glucan yield, while soil nitrogen residue exerted a negative effect. Collectively, these results indicate that enhanced nitrogen uptake improves photosynthetic performance and quality but does not necessarily translate into higher yields. Optimizing nitrogen management is therefore required to balance productivity, profitability, and environmental sustainability.

### 2.6. Multi-Objective Optimization of N Timing and N Rate

**Figure 7:**
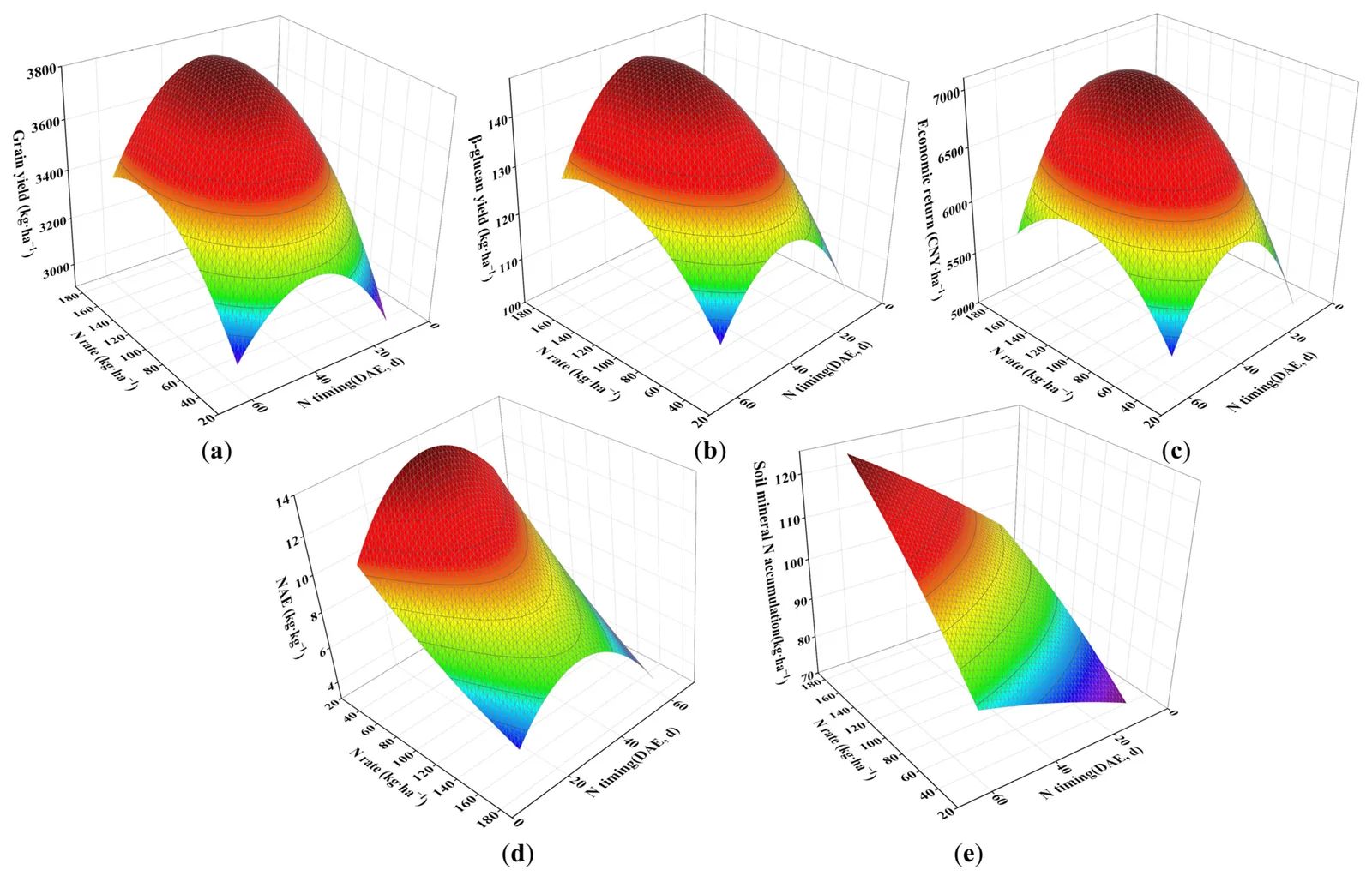
The relation between the *N* rate, *N* timing and each of the following: grain yield (**a**), β-glucan yield (**b**), economic return (**c**), nitrogen agronomic efficiency (**d**), and soil mineral N accumulation (**e**). *N* rate refers to different nitrogen application rates, and *N* timing refers to the timing of topdressing nitrogen fertilizer, which is expressed as the number of days after seedling emergence.

A quadratic regression model was employed to evaluate the effects of nitrogen application rate (R) and nitrogen topdressing timing (T) on key variables (Table S2). The results indicated that all response variables were well described by the regression models, with those for grain yield, NAE, economic return, and soil nitrogen residue reaching highly significant levels (*p* < 0.001) (Figure 7). Grain yield increased with both R and T, but negative quadratic terms suggested the presence of an optimal nitrogen application range. The positive interaction term between R and T further indicated a synergistic effect, enhancing yield beyond individual contributions of R or T. The negative linear coefficient for R indicated that NAE declined under high nitrogen rates, whereas the positive linear and negative quadratic coefficients for T suggested that moderately delaying nitrogen application could improve nitrogen use efficiency, although excessive delay might reduce this benefit. Economic return exhibited a similar “increase-then-decline” trend as yield, significantly influenced by both R and T, with a negative interaction term, implying that combining high nitrogen input with late application was suboptimal for maximizing returns. For soil nitrogen residue, the positive linear terms for R and T and their interaction indicated that higher nitrogen rates and delayed application significantly increased residual soil nitrogen. Based on these findings, multiple regression models were integrated with a genetic algorithm for multi-objective optimization (Table S3). The optimal strategy was a total nitrogen application rate of 140.68 kg·ha^−1^ with topdressing applied 32 days after emergence. This approach resulted in a grain yield of 3757.09 kg·ha^−1^, β-glucan yield of 146.24 kg·ha^−1^, NAE of 8.62, soil nitrogen residue of 104.17 kg·ha^−1^, and an economic benefit of 14,071.02 CNY·ha^−1^, thereby achieving multi-objective goals of reducing fertilizer input, enhancing yield and income, and mitigating environmental pollution.

## 3. Discussion

### 3.1. Optimizing Nitrogen Management for Oat Yield and β-Glucan Improvement

Although oats are tolerant to poor soils, their sensitivity to nitrogen management has received increasing attention in the context of high-yield and high-quality production [20]. The present study demonstrated that when nitrogen fertilizer was topdressed at the T1 stage, β-glucan yield in oats increased continuously with increasing nitrogen application rate. In contrast, topdressing nitrogen fertilizer at the T2 and T3 stages followed a quadratic trend of initial increase followed by decline for grain yield and β-glucan yield as the nitrogen application rate increased, with the optimal nitrogen rate being 140 kg·ha^−1^. These results indicate that an appropriate nitrogen supply can significantly promote grain yield and β-glucan accumulation, whereas excessive nitrogen application stimulates excessive vegetative growth and leads to saturation of physiological processes, ultimately resulting in diminishing returns [21,22]. In particular, excessive nitrogen topdressing during the middle and late growth stages can reduce grain yield and β-glucan yield. This may be associated with intensified competition between vegetative and reproductive growth, which restricts grain filling and grain maturation [23,24].

Because oat nitrogen demand varies across developmental stages, selecting fertilization timing that aligns with crop nutrient requirements is crucial [25]. In this study, nitrogen topdressing at the T2 stage resulted in superior yield and quality compared with T1 and T3. Studies have shown that grain-filling stage development is a critical period for oat β-glucan accumulation, during which the stability and sustainability of nitrogen supply are particularly important for quality development [26]. In this study, moderately delayed nitrogen application optimized nitrogen uptake during the early growth stage, promoted β-glucan accumulation, and avoided both excessive vegetative growths induced by early over-fertilization and yield losses caused by late nitrogen application, thereby simultaneously improving both yield and quality [27]. The interaction between nitrogen topdressing timing and nitrogen application rate further revealed the timing-dependent responses of nitrogen management. This indicates that the integrated effects of nitrogen on oat yield, quality, and nitrogen use efficiency can only be accurately evaluated when nitrogen topdressing timing and application rate are considered jointly [28].

Improving both crop yield and quality is a key goal for nutritional security and sustainable agriculture [29]. Traditional studies often consider a trade-off between quality and yield, especially in cereals [30]. However, our findings indicate that under moderate nitrogen application rates with topdressing at the T2 stage, both oat grain yield and β-glucan content were simultaneously enhanced, achieving a synergistic improvement. This also implies potential positive associations between yield and key nutritional traits, suggesting that optimized fertilization strategies can alleviate the bottleneck of simultaneously enhancing oat yield and quality.

### 3.2. Trade-Off Between Agronomic Efficiency of Nitrogen and Soil Residual Nitrogen

Nitrogen use efficiency is directly related to crop productivity, economic returns and agroecosystem health [31]. Previous studies have shown that excessive nitrogen application not only reduces crop uptake but also markedly decreases NAE [32]. Our results corroborate this pattern: NAE declined significantly with increasing nitrogen input, and the reduction was more pronounced when topdressing was delayed. This suggests that nitrogen losses are amplified under high input, where mismatches between nitrogen supply and crop demand intensify the risk of leaching and accumulation [33]. The underlying mechanism is that excess nitrogen not taken up by crops tends to leach into deeper soil layers [34]. This may explain the significant increase in residual soil nitrogen observed in oat fields with higher nitrogen application rates in this study.

NAE was also significantly affected by the timing of nitrogen topdressing. This outcome likely reflects that T2 fertilization coincides with the peak of crop nitrogen demand, thereby enhancing uptake efficiency and yield response [35]. By contrast, residual soil nitrogen increased significantly with delayed nitrogen application. In line with previous studies, our results confirm that systems with low nitrogen use efficiency tend to result in substantial accumulation of residual nitrogen in the soil profile [36]. Although such residual nitrogen may partially benefit subsequent crops, it nonetheless poses potential environmental risks [37]. PLS-PM analysis further revealed the regulatory pathways among variables. Nitrogen application rate and topdressing timing indirectly influenced NAE and residual soil nitrogen through their effects on crop nitrogen uptake and nitrogen use efficiency. Soil residual nitrogen, in turn, suppressed resource use efficiency and reduced economic returns. Moreover, crop nitrogen utilization efficiency showed a negative association with NAE, while NAE exerted a significant positive effect on profitability. Collectively, these findings highlight a typical “productivity–environment” trade-off in agricultural systems: enhancing nitrogen use efficiency requires balancing productivity gains with the need to mitigate environmental burdens [38].

### 3.3. Physiology and Carbon–Nitrogen Accumulation Underpin Oat Responses to Nitrogen Application Strategies

The synchrony between nitrogen supply and crop demand directly determines the overall efficiency of nitrogen metabolism, photosynthetic physiology, and biomass production [39]. In the present study, leaf nitrogen metabolic enzyme activities under 105–175 kg·ha^−1^ N were significantly higher than those under low N treatments. Among different *N* topdressing timings, these activities under the T2 treatment were significantly higher than those under T1 and T3. These results suggest that an appropriate nitrogen supply effectively sustains physiological activities related to nitrogen metabolism. Moreover, moderate *N* topdressing, which coincides with the peak nutrient demand of oats, further enhances the activities of key enzymes involved in nitrogen assimilation and improves the nitrogen utilization efficiency [26,40]. Meanwhile, higher leaf nitrogen metabolic enzyme activities generally indicate stronger nitrogen uptake, assimilation, and utilization capacity, allowing plants to use available soil nitrogen more efficiently and thereby reducing excessive nitrogen accumulation in soil [41]. This is also consistent with the highly significant positive correlation between soil residual nitrogen and nitrogen metabolic enzyme activities observed in this study.

Flag leaf SPAD and NBI values are key indicators that directly reflect leaf nitrogen status, chlorophyll content, and photosynthetic potential in cereal crops, and their dynamic changes can be used to evaluate the effectiveness of nitrogen management [42]. The period from jointing to grain filling is a critical transition stage from vegetative to reproductive growth in oats, which is sensitive to both the timing and rate of nitrogen supply [43]. The results of our study showed that moderate nitrogen topdressing (T2) maintained higher SPAD and NBI values at the heading and grain-filling stages. This regulatory pattern is consistent with findings in wheat, confirming that matching nitrogen topdressing timing with crop growth stages is a core cultivation measure to sustain leaf photosynthetic activity and ensure grain filling [44]. Regarding nitrogen application rate, SPAD and NBI values increased with increasing nitrogen supply, indicating that higher nitrogen input significantly improved leaf nitrogen status, chlorophyll content, and nitrogen balance index, thereby enhancing photosynthetic performance [45]. However, no significant differences in SPAD and NBI values were observed among high-nitrogen treatments, suggesting that nitrogen utilization in oats had reached a physiological saturation point.

Improved leaf photosynthetic efficiency is ultimately directly manifested as changes in plant dry matter and nitrogen accumulation [46]. Regarding nitrogen topdressing timing, dry matter and nitrogen accumulation under moderate topdressing (T2) were significantly higher than those under early (T1) or late (T3) topdressing. This trend was highly consistent with the earlier variations in SPAD, NBI, and nitrogen metabolic enzyme activities, further verifying the core theory that synchronized nutrient supply and demand can simultaneously enhance plant biomass synthesis and nutrient uptake by strengthening photosynthesis and nitrogen metabolism [47]. With respect to nitrogen application rate, obvious differences in accumulation patterns were observed among different topdressing timings. Under early topdressing (T1), dry matter and nitrogen accumulation increased continuously with increasing nitrogen rate, but the final accumulation level remained relatively low. In contrast, under T2 and T3 treatments, both indices showed a quadratic response, reaching the maximum at N4 and declining under excessive nitrogen application (N5). This is consistent with the concept of a physiological saturation point for crop nitrogen uptake, whereby excessive nitrogen input may limit root nitrogen acquisition and metabolic activity, thereby restricting further increases in nitrogen accumulation efficiency [48]. Among all treatment combinations, the T2N4 treatment achieved significantly higher dry matter and nitrogen accumulation than most other treatments, further indicating that moderate topdressing combined with an appropriate nitrogen rate represents the optimal management strategy for maximizing both dry matter production and nitrogen uptake in oats.

### 3.4. Integrated Trade-Offs and Optimal Strategy Recommendations: Balancing Yield, Quality, Efficiency, and Environment

In oat production, achieving a coordinated balance among high yield, efficient resource use, superior quality, and environmental sustainability is central to formulating effective nitrogen management strategies. There is still a lack of quantitative decision-making frameworks suitable for coarse grain production. Fertilization recommendations mostly rely on experience, making it difficult to meet the theoretical requirements for precision and sustainable production. By integrating multiple regression models with the NSGA-II algorithm, the multi-objective optimization approach not only quantitatively captures the effects of nitrogen variables on agronomic and environmental indicators but also circumvents the subjectivity inherent in expert-weighting methods, thereby enhancing the objectivity and robustness of optimization outcomes. In this study, based on the NSGA-II multi-objective optimization algorithm, we integrated key indicators including grain yield, NAE, β-glucan yield, economic return, and soil nitrogen residue into the NSGA-II multi-objective optimization framework, systematically evaluated synergies and trade-offs across different nitrogen rate and topdressing timing combinations, and obtained satisfactory model fitting results.

This method overcomes the limitations of the traditional single-objective optimization framework and provides a rigorous and feasible model for precision nitrogen management. However, several limitations should be acknowledged. The model was constructed based on field experimental data, and its prediction and optimization performance strongly depends on the experimental environment, sample size, and regional site conditions. Thus, its adaptability to different soil types, climatic conditions, and cultivation systems across ecological regions is limited. Meanwhile, the NSGA-II algorithm in this study focuses on the optimization of two core variables: nitrogen application rate and topdressing timing. It does not incorporate interannual climatic fluctuations such as precipitation and temperature, limiting the dynamic adjustment of optimization parameters. The lack of coupling with crop growth models and real-time meteorological data limits its adaptability to complex and variable field production conditions. To address these limitations, future studies can further improve the system by integrating crop growth models, adding multi-source environmental data, and including additional objective indicators. This will help establish a more universally applicable dynamic optimization framework and advance both the theoretical and practical implications of efficient nitrogen management in minor grain crops.

## 4. Materials and Methods

### 4.1. Study Site Description

**Figure 8:**
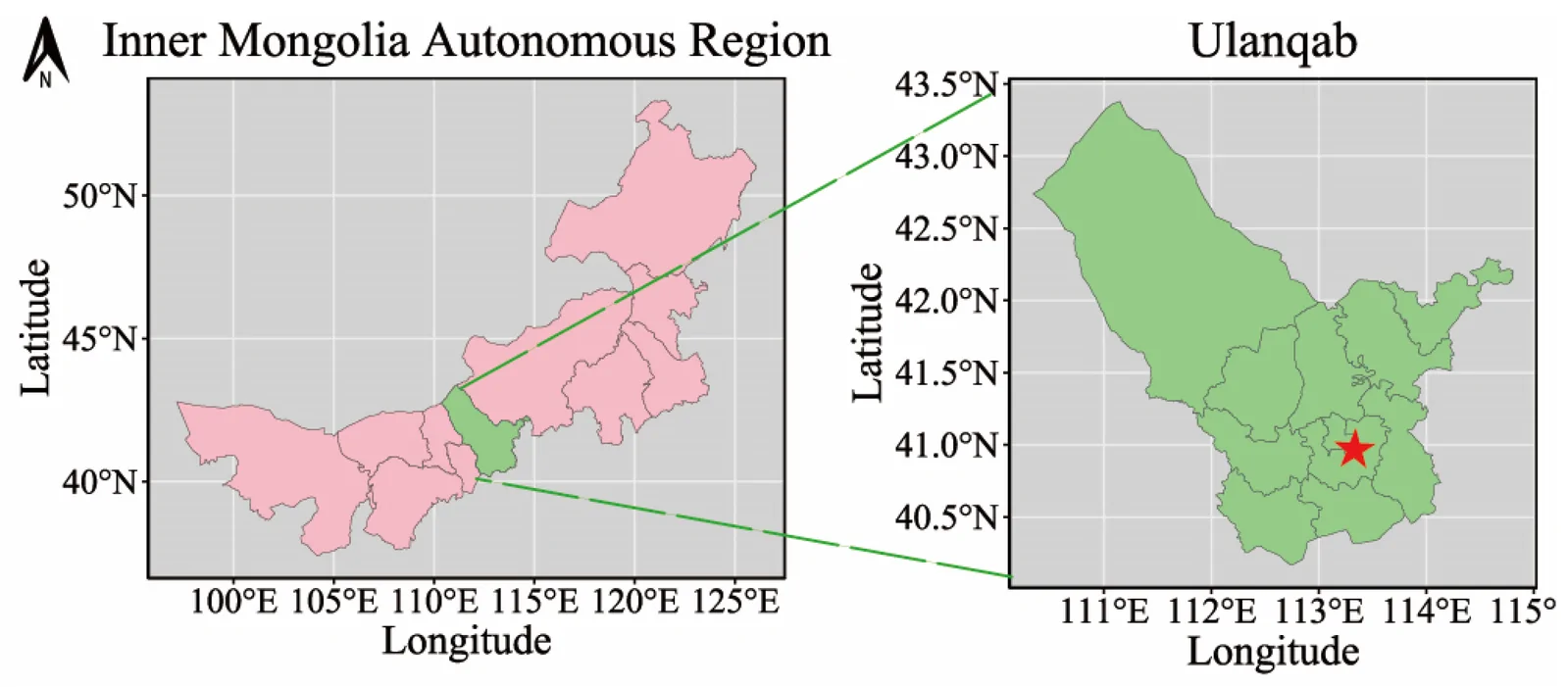
Location map of the test site. Red star indicates the experimental site in the figure caption. Red star indicates the experimental site in the figure caption.

**Figure 9:**
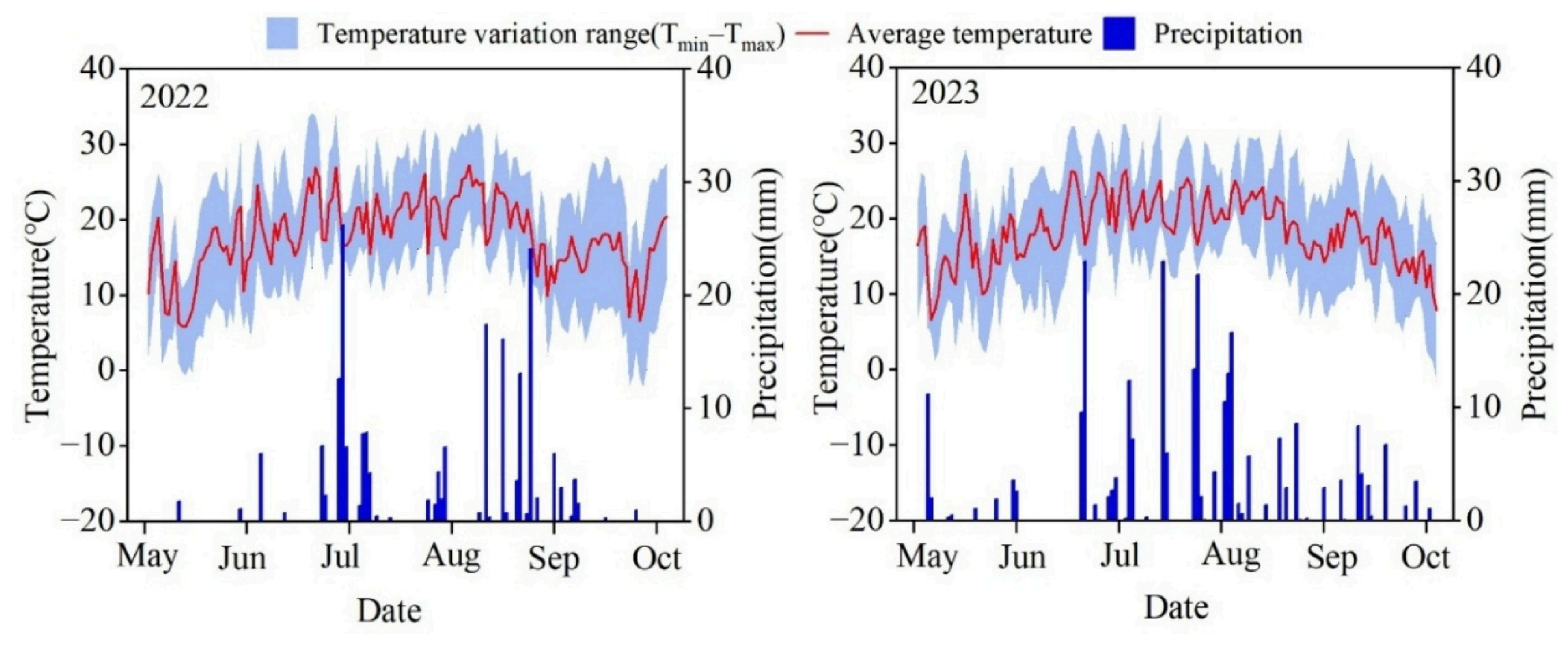
Temperature variation range, average temperature and precipitation during the oat growing periods in 2022 and 2023.

The field experiment was conducted in 2022–2023 at the Ulanqab Academy of Agricultural and Animal Husbandry Sciences, Inner Mongolia Autonomous Region, China. The experimental site was located in Pingdiquan Town, Chahar Right Front Banner, Ulanqab City (Figure 8), situated on the southern slope of the Yinshan Mountains (113.11° E, 40.92° N) in the agro-pastoral ecotone of northern China. The area has a temperate continental monsoon climate and is representative of typical semi-arid agricultural regions. Annual precipitation is unevenly distributed, with approximately 60–70% falling between July and September, and the frost-free period is about 130 days. The temperature and precipitation during the experimental period are shown in Figure 9. The soil at the experimental site is classified as chestnut soil (Haplic Kastanozem, FAO classification). Before sowing, the soil contained 11.69 g·kg^−1^ organic matter, 1.26 g·kg^−1^ total nitrogen, 97.10 mg·kg^−1^ alkali-hydrolyzable nitrogen, 14.12 mg·kg^−1^ available phosphorus, and 129.02 mg·kg^−1^ available potassium, with a pH of 7.79.

### 4.2. Experimental Design and Field Management

A randomized complete block design was adopted for a two-factor experiment. Factor A consisted of three nitrogen topdressing timings: tillering stage (T1), jointing stage (T2), and heading stage (T3). Factor B comprised five nitrogen application rates: 35 kg·ha^−1^ (N1), 70 kg·ha^−1^ (N2), 105 kg·ha^−1^ (N3), 140 kg·ha^−1^ (N4), and 175 kg·ha^−1^ (N5). A zero-nitrogen control treatment (N0, no nitrogen application throughout the growth period) was included as the reference for calculating nitrogen agronomic efficiency. Before sowing, all plots received 80 kg·ha^−1^ of diammonium phosphate as a basal fertilizer. The remaining nitrogen was applied as urea at the designated topdressing stages by broadcasting between rows. Each treatment was replicated three times, resulting in a total of 45 randomly arranged plots. Each plot measured 25 m^2^ (5 m × 5 m).

The oat cultivar Bayou 14, the dominant local variety, was used in this study. Sowing was conducted on 12 May 2022 and 13 May 2023, and harvest was carried out on 28 August 2022 and 2 September 2023. A wide-strip sowing method was adopted with a row spacing of 30 cm, a strip width of 8 cm, and a seeding rate of 180 kg·ha^−1^. Other field management practices followed local oat production protocols. No supplementary irrigation was implemented throughout the growing seasons, and soil moisture was solely dependent on natural precipitation.

### 4.3. Methods for Field Investigation

#### 4.3.1. Grain Yield and β-Glucan Yield

At physiological maturity in each plot, a uniform 3 m^2^ area of oat plants was harvested manually. The oat panicles were air-dried, threshed, cleaned, and weighed. In China, a grain moisture content of 14% is the standard for grain storage and marketing; therefore, oat grain yield was adjusted to this moisture content.

The β-glucan content in oat grains was determined using a Megazyme^®^ mixed-linkage β-glucan assay kit (Megazyme, Bray, Ireland), following the manufacturer’s instructions [8]. The β-glucan yield was then calculated using the following equation:

BGY = BGC × GY/100(1)

BGY, BGC and GY refer to β-glucan yield (kg·ha^−1^), β-glucan content (%) and grain yield of oats (kg·ha^−1^), respectively.

#### 4.3.2. Agronomic Efficiency of Nitrogen (NAE)

NAE was calculated as the increase in grain yield per unit of applied nitrogen, using the N0 control (no N application) as the reference [49].

(2) $$\mathrm{NAE}=\frac{{\mathrm{GY}}_{\mathrm{NA}}-{\mathrm{GY}}_{N0}}{\mathrm{ANA}}$$

GY_NA_, GY_N0_ and ANA refer to grain yield (kg·ha^−1^) in the nitrogen-applied area (kg·ha^−1^), grain yield in the no-nitrogen-applied area (kg·ha^−1^) and amount of nitrogen applied (kg·ha^−1^).

#### 4.3.3. Soil Mineral Nitrogen Accumulation Amount (SNA)

After oat harvest, soil bulk density was determined at three random locations in each plot using the ring knife method. Soil samples were collected from three layers (0–20 cm, 20–40 cm, and 40–60 cm) using a soil auger. Visible plant residues and roots were removed from the samples, which were then sieved through a 2 mm mesh and stored at 4 °C until analysis. Soil ammonium (NH_4_^+^-N) and nitrate (NO_3_^−^-N) contents [50] were determined using a flow injection analyzer (FIAstar 5000, FOSS, Hilleroed, Denmark).

(3) $$S\mathrm{NA}=C\;\times \;H\;\times \;\frac{B}{10}$$

SNA represents soil mineral nitrogen accumulation (kg·ha^−1^), C is the concentration of soil nitrate-N and ammonium-N (mg·kg^−1^), H is the thickness of the soil layer (cm), and B is the soil bulk density (g·cm^−3^).

#### 4.3.4. Economic Benefit

Economic benefits [51] were primarily calculated as the difference between total revenue and total input costs. Total revenue was obtained by multiplying oat grain yield by its unit price. Total input costs included fertilizer expenses, as well as costs for sowing, weed control, and harvesting. In this study, the prices were set as follows: urea at 2500 CNY·t^−1^, diammonium phosphate (DAP) at 3710 CNY·t^−1^, oat seed at 7.5 CNY·kg^−1^; sowing costs (excluding fertilizer and seed inputs) were 800 CNY·ha^−1^, weed control costs 300 CNY·ha^−1^, and harvesting costs 1200 CNY·ha^−1^. The main product was oat grain, priced at 5 CNY·kg^−1^.

(4) ER=TR−TI

ER, TR, and TI represent Economic Return, Total Revenue, and Total Input, respectively.

#### 4.3.5. DM Accumulation and N Accumulation

At oat maturity, 30 plants were randomly sampled from each plot. The plants were separated into stems (stalks and leaf sheaths), leaves (leaf blades), and panicles (rachis, glumes, and grains) for biomass determination. Samples were oven-dried to constant weight and weighed to obtain dry matter (DM) weight. The DM accumulation per hectare was then calculated based on the pre-surveyed plant density. The dried samples were ground and sieved, and the nitrogen content of the oat plants was determined using the Kjeldahl method. Nitrogen accumulation in the plant population was calculated according to the plant density per hectare.

#### 4.3.6. Chlorophyll Content (SPAD) and Nitrogen Balance Index (NBI)

At the jointing, heading, and grain-filling stages, nine uniformly grown oat plants were selected from each plot. The SPAD and NBI of leaves were measured using a portable polyphenol-chlorophyll meter (Dualex-3, Force-A, Orsay, France). The final value was the average of the measured values.

#### 4.3.7. The Activities of Nitrate Reductase (NR) and Glutamine Synthetase (GS)

At the grain-filling stage, nine uniformly vigorous oat plants were selected from each plot. The flag leaves were washed with distilled water, blotted dry with absorbent paper, and immediately stored in liquid nitrogen. The activities of NR and GS were determined using enzyme-linked immunosorbent assay (Infinite M200, Tecan, Männedorf, Switzerland).

#### 4.3.8. Model Development

To quantify the effects of nitrogen topdressing rate and topdressing timing on various agronomic traits and to provide mathematical models for subsequent multi-objective optimization, quadratic polynomial multiple linear regression models were developed for multiple response variables [19]. Each model used nitrogen rate (X_1_) and nitrogen topdressing timing (X_2_) as independent variables. The response variables included grain yield, nitrogen agronomic efficiency, economic return, soil mineral nitrogen residue (SNA), and β-glucan yield. The general form of the model is as follows:

(5) $$Y\;=\;\beta_{0}\;+\;\beta_{1}X_{1}\;+\;\beta_{2}X_{2}\;+{\;\beta }_{3}X_{1}^{2}\;+{\;\beta }_{4}X_{2}^{2}\;+{\;\beta }_{5}X_{1}X_{2}\;+\;\varepsilon$$

where Y represents the corresponding response variable, β_0_ is the intercept, β_1_ to β_5_ are the regression coefficients, and ε is the error term.

To ensure model reliability, distribution tests and necessary data transformations were performed on all variables prior to modeling. Multicollinearity among variables was also checked. All models met the fundamental assumptions of linear regression and demonstrated good fit and predictive performance. The resulting regression models were used as mathematical expressions of objective functions in the subsequent NSGA-II multi-objective optimization algorithm to support decision-making.

#### 4.3.9. NSGA-II for Multi-Objective Optimization

The NSGA-II optimization was implemented using the mco package 1.17 in R statistical software (version 4.4.2). The complete analytical procedure followed the step-by-step workflow described below.

##### Construction of Objective Functions Based on Regression Models

In this study, a multi-objective optimization problem was formulated for nitrogen management strategies in oat production with five objectives: yield (f_1_), nitrogen agronomic efficiency (f_2_), net income (f_3_), β-glucan yield (f_4_), and soil nitrogen residue (f_5_). Nitrogen rate (x_1_) and nitrogen topdressing timing (x_2_) were selected as decision variables. The objective functions were constructed based on quadratic polynomial regression models as follows:

(6) $$\left\{\begin{array}{c} maxf_{1}(x_{1},x_{2})=a_{1}+b_{1}x_{1}+c_{1}x_{2}+d_{1}x_{1}^{2}+e_{1}x_{2}^{2}+k_{1}x_{1}x_{2}\\ maxf_{2}(x_{1},x_{2})=a_{2}+b_{2}x_{1}+c_{2}x_{2}+d_{2}x_{1}^{2}+e_{2}x_{2}^{2}+k_{2}x_{1}x_{2}\\ maxf_{3}(x_{1},x_{2})=a_{3}+b_{3}x_{1}+c_{3}x_{2}+d_{3}x_{1}^{2}+e_{3}x_{2}^{2}+k_{3}x_{1}x_{2}\\ maxf_{4}(x_{1},x_{2})=a_{4}+b_{4}x_{1}+c_{4}x_{2}+d_{4}x_{1}^{2}+e_{4}x_{2}^{2}+k_{4}x_{1}x_{2}\\ minf_{5}(x_{1},x_{2})=a_{5}+b_{5}x_{1}+c_{5}x_{2}+d_{5}x_{1}^{2}+e_{5}x_{2}^{2}+k_{5}x_{1}x_{2} \end{array}\right.$$

(7) $${\mathrm{IR}}_{i}\;\leq \;x_{i}\;\leq {\;\mathrm{UR}}_{i}$$

where IR_i_ and UR_i_ are the lower and upper bound constraints of the variable xi, respectively.

Based on this, the NSGA-II was further employed to perform multi-objective optimization of nitrogen management strategies, aiming to maximize yield, nitrogen agronomic efficiency, net income, and β-glucan yield, while minimizing soil nitrogen residue.

##### Definition of Decision Variables and Boundary Constraints

Nitrogen topdressing rate and nitrogen topdressing timing were set as the two decision variables for optimization. The lower and upper bounds of each variable were defined according to the minimum and maximum values of the experimental treatments, to restrict the search space within the range of observed data and avoid unreliable extrapolation beyond measured conditions.

##### Algorithm Parameterization and Execution

The NSGA-II algorithm was configured with standard real-coded evolutionary operators, including simulated binary crossover and polynomial mutation, with a distribution index of 20 for both operators. The population size was set to 100 individuals, and the maximum number of evolutionary generations was set to 500 to ensure sufficient convergence of the Pareto front.

##### Generation of the Pareto Optimal Solution Set

During each generation, all individuals in the population underwent fitness evaluation via fast nondominated sorting and crowding distance calculation. Fast nondominated sorting ranked individuals into hierarchical Pareto fronts based on their nondomination status, while crowding distance maintained population diversity to prevent premature convergence to local optima. An elitist strategy was applied to retain high-quality solutions from both parent and offspring populations across generations. After 500 generations of evolution, the final output was a set of nondominated solutions forming the Pareto optimal front. Each solution in this set represents a Pareto optimal nitrogen management strategy, where no single objective can be improved without degrading at least one other objective.

##### Selection of the Comprehensive Recommended Optimal Strategy

To assist decision-making, the optimal solution was selected from the Pareto set based on a comprehensive scoring method, where weights were assigned according to the relative importance of yield, nitrogen agronomic efficiency, net income, soil nitrogen residue, and β-glucan yield. The optimization results offer quantitative support and feasible pathways for sustainable and efficient nitrogen management.

### 4.4. Data Analysis

Data variance analysis was performed using SAS version 9.4 (SAS Institute Inc., Cary, NC, USA). A two-way ANOVA was used to determine the effects of *N* timing, *N* rate, and their interaction on all indices. *N* timing and *N* rate were treated as fixed effects, while replication was considered a random effect. When the interaction was not significant, only the main effects of *N* timing and *N* rate were analyzed, and multiple comparisons among treatment combinations were not performed to ensure valid statistical inference; when the *N* timing × *N* rate interaction was significant (*p* < 0.05), simple effects of each treatment combination were further analyzed. Multiple comparisons of treatment means were conducted using Fisher’s least significant difference (LSD) test; differences were considered significant at *p* < 0.05 and highly significant at *p* < 0.01. Partial least squares path models (PLS-PMs) were constructed using the plspm package 0.6.0 in R software (version 4.4.2, R Core Team, Vienna, Austria) to analyze the relationships between crop growth traits, grain yield, economic return, and soil nitrogen residue under different nitrogen management regimes. Multiple regression models were developed using R software, and the elitist nondominated sorting genetic algorithm (NSGA-II) was implemented using the mco package in R software for multi-objective optimization. All figures were generated using Origin 2024 (OriginLab Corporation, Northampton, MA, USA) and R software. Specifically, physiological and agronomic parameters (dry matter and nitrogen accumulation, SPAD, NBI, enzyme activities, etc.), correlation diagrams, and multiple regression model figures under different treatments were plotted using Origin 2024, whereas chord diagrams were visualized using the circlize package 0.4.18 and tidyverse package 2.0.0 in R software.

## 5. Conclusions

This study demonstrates that topdressing at the jointing stage combined with medium-to-high nitrogen application rates (105–140 kg·ha^−1^) can precisely match the peak nitrogen demand of oats, enhance leaf nitrogen metabolism enzyme activities and photosynthetic capacity, promote the efficient accumulation of dry matter and nitrogen, and thereby achieve the synergistic improvement of oat grain yield, β-glucan content and economic return. However, with increasing nitrogen application rate and excessive delay in topdressing time, the agronomic efficiency of nitrogen fertilizer decreases significantly, and soil nitrogen residue increases concurrently. There is a significant trade-off relationship between farmland nitrogen input, crop nitrogen utilization and soil nitrogen residue. Based on multiple regression models and the NSGA-II algorithm, multi-objective optimization was carried out to identify the optimal nitrogen application scheme, taking into account multiple benefits: a total nitrogen application rate of 140.68 kg·ha^−1^ and nitrogen topdressing 32 days after seedling emergence. This scheme can minimize the risk of soil nitrogen residual accumulation while ensuring oat yield, quality and economic benefits. This study constructed a multi-objective optimization system for oat nitrogen management suitable for the agro-pastoral ecotone of northern China, which provides a theoretical basis and practical approaches for the synergistic promotion of high-yield, high-quality oat production and ecological conservation in the region.

## Figures and Tables

**Table 1:** Effects of nitrogen topdressing timing and rate on oat grain yield, β-glucan yield, and nitrogen agronomic efficiency (NAE) in 2022 and 2023.

Treatment		Grain Yield (t·ha^−1^)		β-Glucan Yield (kg·ha^−1^)		NAE (kg·kg^−1^)	
*N* Timing	*N* Rate	2022	2023	2022	2023	2022	2023
T1	N1	2.92 g	3.02 h	95.61 f	118.76 h	9.24 de	9.89 Ba
	N2	3.11 ef	3.26 fg	107.49 de	130.54 g	7.35 def	8.41 Bb
	N3	3.26 bcde	3.39 ef	110.98 cde	136.65 efg	6.29 gh	6.84 Bc
	N4	3.35 b	3.57 cd	114.46 c	145.38 cd	5.42 hi	6.42 Bd
	N5	3.32 bc	3.50 de	120.95 b	148.89 c	4.17 i	4.76 Be
T2	N1	3.12 def	3.14 gh	106.06 e	130.78 g	15.13 a	13.39 Aa
	N2	3.42 b	3.46 de	121.05 b	144.37 cde	11.74 bc	11.27 Ab
	N3	3.69 a	3.85 ab	126.95 a	165.01 ab	10.43 cd	11.21 Ac
	N4	3.76 a	3.90 a	129.45 a	169.80 a	8.34 ef	8.79 Ad
	N5	3.66 a	3.73 bc	124.44 ab	160.36 b	6.07 gh	6.06 Ae
T3	N1	3.04 fg	3.05 h	99.17 f	120.95 h	12.69 b	10.50 Ba
	N2	3.18 cdef	3.24 fg	106.51 de	133.18 fg	8.39 ef	8.14 Bb
	N3	3.30 bc	3.36 ef	111.79 cd	139.33 def	6.69 fgh	6.57 Bc
	N4	3.34 bc	3.49 de	111.78 cd	145.19 cd	5.33 hi	5.82 Bd
	N5	3.28 bcd	3.46 de	109.98 cde	143.15 cde	3.94 i	4.52 Be
Variance analysis							
*N* timing		**	**	**	**	**	**
*N* rate		**	**	**	**	**	**
*N* timing × *N* rate		*	*	**	*	*	ns

Notes: Different lowercase letters indicate significant differences among the 15 treatment combinations when the *N* timing × *N* rate interaction is significant (*p* < 0.05). When the *N* timing × *N* rate interaction is not significant, different uppercase letters indicate significant differences among *N* timing factor main effects, and different lowercase letters indicate significant differences among *N* rate main effects (*p* < 0.05). * Significant at *p* < 0.05; ** Significant at *p* < 0.01; ns, not significant at *p* > 0.05. T1, T2, and T3 represent three different topdressing timing treatments, respectively; N1–N5 represent five different nitrogen application rate treatments, respectively.

**Table 2:** Effects of different *N* timing and *N* rate treatments on economic return.

Treatment		Total Input (×10^3^ CNY·ha^−1^)		Total Revenue (×10^3^ CNY·ha^−1^)		Economic Return (×10^3^ CNY·ha^−1^)	
*N* Timing	*N* Rate	2022	2023	2022	2023	2022	2023
T1	N1	4.14	4.14	14.60 g	15.08 h	10.46 f	10.95 h
	N2	4.33	4.33	15.55 ef	16.30 fg	11.22 def	11.97 ef
	N3	4.52	4.52	16.30 bcde	16.94 ef	11.78 de	12.42 cde
	N4	4.71	4.71	16.75 b	17.85 cd	12.04 cd	13.14 bc
	N5	4.90	4.90	16.60 bc	17.50 de	11.70 de	12.60 cde
T2	N1	4.14	4.14	15.62 def	15.69 gh	11.48 de	11.56 fhg
	N2	4.33	4.33	17.08 b	17.29 de	12.76 bc	12.97 bcd
	N3	4.52	4.52	18.45 a	19.23 ab	13.93 a	14.71 a
	N4	4.71	4.71	18.81 a	19.51 a	14.10 a	14.79 a
	N5	4.90	4.90	18.29 a	18.65 bc	13.38 ab	13.75 b
T3	N1	4.14	4.14	15.19 fg	15.19 h	11.06 ef	11.05 gh
	N2	4.33	4.33	15.91 cdef	16.20 fg	11.58 de	11.87 efg
	N3	4.52	4.52	16.49 bc	16.80 ef	11.97 cd	12.28 efg
	N4	4.71	4.71	16.70 bc	17.43 de	11.99 cd	12.71 cde
	N5	4.90	4.90	16.42 bcd	17.30 de	11.52 de	12.40 cde
Variance analysis							
*N* timing		-	-	**	**	**	**
*N* rate		-	-	**	**	**	**
*N* timing × *N* rate		-	-	*	*	*	*

Notes: The different lowercase letters in the same column indicate significant differences (*p* < 0.05). * Significant at *p* < 0.05; ** Significant at *p* < 0.01. T1, T2, and T3 represent three different topdressing timing treatments, respectively; N1–N5 represent five different nitrogen application rate treatments, respectively; CNY represents Chinese yuan.

## Data Availability

The original contributions presented in this study are included in the article. Further inquiries can be directed to the corresponding author.

## Supplementary Materials

The following supporting information can be downloaded at: https://www.mdpi.com/article/10.3390/plants15203127/s1, Table S1: Two-factor analysis of *N* timing and *N* rate on NBI and SPAD value from 2022 to 2023. Table S2: Regression equations between *N* rate and *N* timing and grain yield, β-Glucan yield, NAE, economic return and soil mineral N accumulation (SNA); Table S3: Optimal nitrogen application scheme based on the multi-objective optimization model.
