# Supplementary material for: Multi-Objective Optimization of Nitrogen Management for Oat Production in the Agro-Pastoral Ecotone of China Using NSGA-II to Balance Yield, Quality, Nitrogen Use Efficiency and Environmental Sustainability
Source: Plants (Basel). 2026 Oct 13;15(20):3127. doi: 10.3390/plants15203127 (PMC13708557; doi:10.3390/plants15203127)
Supplement: Supplementary file 1 [file plants-15-03127-s001.zip › plants-4583183-supplementary.pdf]

Multi-Objective Optimization of Nitrogen Management for Oat Production in the Agro-Pastoral Ecotone of China Using NSGA-II to Balance Yield, Quality, Nitrogen Use Efficiency and Environmental Sustainability

Ying Wang[1], Xue Han [2], Yanli Zhang [3], Xiaorong Wu [1]* and Lijun Li [1,]*

[1] College of Agronomy, Inner Mongolia Agricultural University, Huhhot 010019, China; nxywy123@imau.edu.cn (Y.W.)
[2] Baotou Institute of Agriculture and Animal Husbandry Science and Technology, Baotou, 014000, Inner Mongolia, China
[3] Institute of Resources, Environment and Sustainable Development, Inner Mongolia Academy of Agricultural & Animal Husbandry Sciences, Hohhot 010031, China;
* Correspondence: lijun-li@imau.edu.cn (L.L.); shendu2012126636@163.com (X.W.).

Table S1 Two-factor analysis of N timing, and N rate on NBI and SPAD value from 2022 to 2023.

Treatment		2022			2023		
N timing	N rate	Jointing	Heading	Filling	Jointing	Heading	Filling
NBI							
N timing		**	**	**	**	**	**
N rate		**	**	**	**	**	**
N timing × N rate		**	**	ns	**	ns	ns
SPAD							
N timing		**	**	**	**	**	**
N rate		**	**	**	**	**	**
N timing × N rate		*	ns	ns	ns	ns	*

Note: The sample size n=45, *, ** and ns indicated significant levels at $P< 0.05$, $P< 0.01$ and $P \geq 0.05$, respectively. N rate: nitrogen application rate, and N timing: timing of topdressing nitrogen fertilizer; NBI: nitrogen balance index; SPAD: chlorophyll content.

Table S2 Regression equations between N rate and N timing and Grain yield, β Glucan yield, NAE, Economic return and soil mineral N accumulation (SNA).

Response variable Y	Regression equation	R^2	F	P
Grain yield/	Y_1 =2219.00+13.0R＋34.04T－0.0378R^2－0.4427T^2＋0.04001RT	0.9170	53.04	***
β Glucan yield	Y_2 =70.2845＋0.5862R＋1.9111T－0.0018R^2－0.0253T^2＋0.0015RT	0.7738	2.87	**
NAE	Y_3 =8.695－0.0534R＋0.3794T+0.000097R^2－0.0049T^2－0.00036RT	0.9334	67.28	***
Economic return	Y_4 = 3160.398＋33.572R＋102.133T–0.1134R^2–1.3282T^2–0.12004RT	0.8633	30.31	***
SNA	Y_5 =59.7630＋0.251R＋0.3410T－0.000407R^2－0.001438T^2＋0.001702RT	0.7969	18.83	***

Note: R and T represent the nitrogen application rate and the timing of topdressing nitrogen fertilizer respectively. NAE: nitrogen agronomic efficiency; SNA: nitrogen agronomic efficiency.

Table S3 Optimal nitrogen application scheme based on the multi-objective optimization model.

	N timing	N rate (kg·ha^{-1})	Yield (kg·ha^{-1})	BGY (kg·ha^{-1})	NAE (kg·ha^{-1})	Soil N residue (kg·ha^{-1})	Economic return (CNY·ha^{-1})
Solutions	32.09	140.68	3757.09	146.24	8.62	104.17	14071.02

Note: Yield: grain yield, BGY: β-glucan yield, NAE: nitrogen agronomic efficiency.
